# Dynamic Acquisition and Loss of Dual-Obligate Symbionts in the Plant-Sap-Feeding Adelgidae (Hemiptera: Sternorrhyncha: Aphidoidea)

**DOI:** 10.3389/fmicb.2017.01037

**Published:** 2017-06-13

**Authors:** Carol D. von Dohlen, Usha Spaulding, Kistie B. Patch, Kathryn M. Weglarz, Robert G. Foottit, Nathan P. Havill, Gaelen R. Burke

**Affiliations:** ^1^Department of Biology, Utah State University, LoganUT, United States; ^2^Agriculture and Agri-Food Canada, OttawaON, Canada; ^3^United States Forest Service, Northern Research Station, HamdenCT, United States; ^4^Department of Entomology, University of Georgia, AthensGA, United States

**Keywords:** bacterial symbionts, complex life cycles, dual symbionts, insects, host alternation, symbiont replacements

## Abstract

Sap-sucking insects typically engage in obligate relationships with symbiotic bacteria that play nutritional roles in synthesizing nutrients unavailable or in scarce supply from the plant-sap diets of their hosts. Adelgids are sap-sucking insects with complex life cycles that involve alternation between conifer tree species. While all adelgid species feed on spruce during the sexual phase of their life cycle, each adelgid species belongs to a major lineage that feeds on a distinct genus of conifers as their alternate host. Previous work on adelgid symbionts had discovered pairs of symbionts within each host species, and unusual diversity across the insect family, but left several open questions regarding the status of bacterial associates. Here, we explored the consistency of symbionts within and across adelgid lineages, and sought evidence for facultative *vs.* obligate symbiont status. Representative species were surveyed for symbionts using 16*S* ribosomal DNA gene sequencing, confirming that different symbiont pairs were consistently present within each major adelgid lineage. Several approaches were used to establish whether symbionts exhibited characteristics of long-term, obligate mutualists. Patterns of symbiont presence across adelgid species and diversification with host insects suggested obligate relationships. Fluorescent *in situ* hybridization and electron microscopy localized symbionts to bacteriocyte cells within the bacteriome of each species (with one previously known exception), and detection of symbionts in eggs indicated their vertical transmission. Common characteristics of long-term obligate symbionts, such as nucleotide compositional bias and pleomorphic symbiont cell shape were also observed. Superimposing microbial symbionts on the adelgid phylogeny revealed a dynamic pattern of symbiont gains and losses over a relatively short period of time compared to other symbionts associated with sap-sucking insects, with each adelgid species possessing an older, “senior” symbiont and a younger “junior” symbiont. A hypothesis relating adelgid life cycles to relaxed constraints on symbionts is proposed, with the degradation of senior symbionts and repeated acquisition of more junior symbionts creating opportunities for repeated colonization of new alternate-conifer hosts by adelgids.

## Introduction

Associations between bacterial symbionts and plant-sap-feeding insects are well documented for many insect groups (Buchner, 1965). Such symbionts are typically housed in a large, abdominal organ (the bacteriome). Most sap-sucking insects harbor a single, ancient symbiont that was acquired in the common ancestor of their lineage, coevolved as the lineage diversified, and was retained almost universally within the lineage (Munson et al., 1991; Thao et al., 2000b; Spaulding and von Dohlen, 2001; Thao and Baumann, 2004; Moran et al., 2005; Kuechler et al., 2013). Genomic and experimental studies have characterized these associations as obligate, mutualistic partnerships, in which the bacteria provides the host with nutrients missing or rare in the insect’s diet (e.g., amino acids, vitamins) (Douglas, 1993; Shigenobu et al., 2000; Douglas et al., 2001; Moran et al., 2003b; Nakabachi et al., 2006; Gunduz and Douglas, 2009; Jiang et al., 2012). In certain sternorrhynchan Hemiptera (i.e., aphids and whiteflies) this original bacterium may be the sole nutrient-providing symbiont (Baumann et al., 1995; Thao and Baumann, 2004), but there are some exceptions (Lamelas et al., 2011; Rao et al., 2015). In other Hemiptera, the universal symbiont is typically joined by a co-obligate partner that was acquired more recently and independently in different host sub-lineages (Thao et al., 2000a, 2002; Moran et al., 2003a; Takiya et al., 2006; Bressan et al., 2009; Gatehouse et al., 2012; Noda et al., 2012; Rosenblueth et al., 2012; Urban and Cryan, 2012). Where co-obligate symbiont roles have been investigated through genomics, contributions of the younger partner are shown to complement capabilities of the original symbiont, particularly where genes involving nutrient synthesis have been lost by the older bacterium (Wu et al., 2006; McCutcheon and Moran, 2007, 2010; McCutcheon et al., 2009; Sloan and Moran, 2012; Bennett et al., 2014). Acquisitions of co-symbionts have been characterized as compensatory events that rescue the partnership from deleterious gene deletions in the original symbiont due to genetic drift (Moran, 1996; Wernegreen, 2002; Bennett and Moran, 2015).

Acquisitions and replacements of obligate symbionts seem to have occurred rarely over the history of a host lineage (Bennett and Moran, 2015). For example, within the ∼260 million-year-old (MYO) Auchenorrhyncha, approximately seven co-symbiont acquisitions or replacements are hypothesized (Bennett and Moran, 2013, 2015). Symbiont turnover appears to be more frequent in Coccoidea (scales, mealybugs) (also > 250 MYO; Vea and Grimaldi, 2016), but the number of events is unclear (Rosenblueth et al., 2012). In general, the prevalence of ancient, near-universal symbionts in many insect lineages suggests strong, ongoing selection to maintain the original symbiont despite deleterious genome decay (Bennett and Moran, 2015).

Adelgids (Sternorrhyncha: Aphidoidea: Adelgidae) constitute an unusual case of high diversity in putatively obligate symbionts. Close relatives of aphids, adelgids are highly species-poor in comparison (∼70 adelgid vs. ∼5000 aphid species) (Blackman and Eastop, 1994; Favret et al., 2015). In contrast to the broad host-plant-family diversity of aphids, adelgids feed exclusively on conifer trees (Pinaceae). All species have host-alternating life cycles, or are recent derivatives of host-alternating species (Havill and Foottit, 2007). Host-alternating cycles encompass initial generations on spruce (*Picea* spp.), and subsequent generations on one of five different alternate-conifer genera (*Abies, Larix, Pinus, Pseudotsuga, Tsuga*) (Annand, 1928). A molecular phylogenetic study resolved five major lineages of adelgids corresponding to these alternate-conifer associations (Havill et al., 2007).

Similar to other sap-feeding hemipterans, adelgids harbor bacterial endosymbionts (Profft, 1937; Steffan, 1976). Recent molecular and microscopy studies elucidated the identity, locations, and structural details of endosymbionts (Toenshoff et al., 2012a,b, 2014; Michalik et al., 2013; von Dohlen et al., 2013). These studies document an unexpected diversity of symbionts: while each adelgid species hosts two putatively obligate symbionts, the pairs of symbionts are different in each major adelgid lineage. These associations suggest that both symbionts have been periodically replaced during the ∼90 MY adelgid history (Toenshoff et al., 2012b, 2014).

The pattern of symbiont diversity in adelgids is markedly different from many other hemipteran lineages, and raises several questions concerning the status of symbionts in relation to adelgid biology. In addition, only one species each from four adelgid lineages and two species from the fifth lineage were sampled in studies so far (Toenshoff et al., 2012a,b, 2014; von Dohlen et al., 2013). Thus, the aim of the present study was to sample a broader phylogenetic sample of adelgid species and populations with the goals of determining (a) the identities of symbionts, (b) the consistency of symbiont composition within the major adelgid lineages, and (c) the likelihood that symbionts are co-obligate, long-term mutualists, versus facultative associates. To accomplish this, we sampled five new species and several new populations of previously sampled species, including geographically distinct populations, and populations on alternate conifer hosts. We amplified and sequenced bacterial 16*S* ribosomal genes, assessed sequence characteristics, and checked for evidence of cospeciation between insects and symbionts. We performed *in situ* hybridizations on selected samples to localize symbionts within insects, and examined ultrastructure of symbionts. Based on our findings, we propose a hypothesis of historical turnover in symbionts, and speculate on why symbiont losses and gains have been comparatively dynamic in adelgids *versus* other sternorrhynchan lineages of similar age or older.

## Materials and Methods

### Taxon Sampling, Gene Amplification, Cloning, and Sequencing

Adelgidae species and populations of both genera, *Adelges* and *Pineus*, were sampled from North America, Europe, Taiwan, and Japan (**Table 1**). These represented five newly sampled species, eight new geographically separated populations of four previously sampled species, and two populations of previously sampled species from alternate-host conifers. Morphological identifications were confirmed by R. G. Foottit, and voucher specimens of all samples were deposited in the Utah State University Insect Collection (Logan, UT, United States), and the Canadian National Collection of Insects (Ottawa, ON, Canada). Identifications were further confirmed with DNA (COI) barcodes (Foottit et al., 2009).

**Table 1:** Collection information for new samples used in this study.

Adelgid species	Voucher ID/CNC#	Stage	Location and date	Host
*Adelges abietis* (Linnaeus, 1758)	00-47	gallicola	United States, MI, Ann Arbor; 09-July-2000	*Picea abies* (gall)
*Adelges abietis* (Linnaeus, 1758)	96EM-0427/CNCHEM012423	gallicola	Canada: PEI; 26-August-1996	*Picea glauca* (gall)
*Adelges cooleyi* (Gillette, 1907)	Ad04-28	gallicola, egg	United States: UT, Logan; June 2004	*Picea pungens* (gall)
*Adelges cooleyi* (Gillette, 1907)	2001EM-0264/CNCHEM039367	gallicola	Canada: AB, Coleman; 29-July-2001	*Picea glauca* (gall)
*Adelges cooleyi* (Gillette, 1907)	2001EM-0693/CNCHEM039883	gallicola	Canada: BC, Mount Robson Provincial Park; 6-August-2001	*Picea glauca* (gall)
*Adelges cooleyi* (Gillette, 1907)	2001EM-0910/CNCHEM040090	gallicola	Canada: AB, Castle Mountain Resort; 11-August-2001	*Picea glauca* (gall)
*Adelges japonicus* (Monzen, 1929)	94-81	fundatrix	Japan: Nopporo, Ebetsu-shi; 18-September-1994^1^	*Picea jezoensis*
*Adelges lariciatus* (Patch, 1909)	2001EM-801/CNCHEM040004	gallicola	Canada: AB, Edson; 1-August-2001	*Picea glauca* (gall)
*Adelges laricis* (Vallot, 1836)	Ad05-04	exulis, egg	Canada: ON, Ottawa; June 2005	*Larix decidua*
*Adelges tsugae* (Annand, 1924)	15-027.03	exulis	Taiwan: Nantou, Yuan Feng; 12-April-2015^2^	*Tsuga chinensis*
*Pineus coloradensis* (Gillette, 1907)	98EM-0005/CNCHEM025795	exulis	Canada: ON, Ottawa; 15-April-1998	*Pinus nigra*
*Pineus pini* (Goeze, 1778)	02-02	exulis	United States: UT, Logan; 23-April-1998	*Pinus mugo*
*Pineus similis* (Gillette, 1907)	02-53	gallicola	Canada: BC, Saanichton^3^; 17-June-2002	*Picea sitchensis*
*Pineus similis* (Gillette, 1907)	2000EM-0193/ CNCHEM032874	gallicola	United States: ID, Priest Lake Road; 12-July-2000	*Picea engelmanni*
*Pineus similis* (Gillette, 1907)	98EM-0349/CNCHEM026157	exulis	Canada: BC, Martha Creek Provincial Park; 11-August-1998	*Pinus monticola*
*Pineus similis* (Gillette, 1907)	Ad05-05	gallicola, egg	Canada: BC, Saanichton; 12-July-2005^3^	*Picea sitchensis*

*^1^Collected with S. Akimoto and Y. Yamaguchi.*

*^2^Collected by S. Shiyake.*

*^3^Collected by R. Bennett.*

Molecular methods followed those in von Dohlen et al. (2013). Briefly, genomic DNA was extracted from whole insects. Polymerase chain reaction (PCR) was used to amplify bacterial 16*S* rRNA genes using the general eubacterial primers, 10f and 1507r, or 27f and 1495r. However, these general primer combinations failed to amplify *Annandia* 16*S* rRNA genes from *Pineus* spp. Instead, these samples were amplified with eubacterial primers 341f and 1507r. After determining a partial 16*S* sequence of *Annandia* from *Pineus similis*, specific primers PinGam1_61f and PinGam1_1161r (**Table 2**) were designed to amplify the 5′ end of this molecule in *P. similis* (sample 02-53). In addition, specific primers ATGamC62f and ATGamC1161r (**Table 2**) were designed from a previously obtained sequence of *A. tsugae* (sample NH04-36 from Japan, on *Tsuga diversifolia*; GenBank #KC331955) to amplify the 5′ end of 16*S* for this species. PCR products were sub-cloned, and 10 or more clones were sequenced in one direction to check for unique sequences. Single clones representing unique sequences were sequenced fully with the same primers used for PCR and internal primers 766f, 570r, and 810r (von Dohlen et al., 2013). All 16*S* rDNA sequences were submitted to GenBank under the following accessions: MF077637–MF077640 and MF098761 (‘*Ca*. Annandia pinicola’), MF077633–MF077636 (‘*Ca*. Gillettellia cooleyia’), MF077641–MF077645 (‘*Ca*. Hartigia pinicola’), MF108835-MF108838 (‘*Ca*. Profftia spp.’), MF098762 (‘*Ca*. Pseudomonas adelgestsugas from Taiwan), MF063340-MF063348 (‘*Ca*. Vallotia spp.’).

**Table 2:** New oligonucleotide primers and probes used in this study.

Probe or primer	Sequence (5′ to 3′)	Target species
**Primers**		
ATGamC62f	CTG TTT ATT TTA AAT AAT AG	*‘Ca.* Annandia’ in *Adelges tsugae*
ATGamC1161r	AAT TAT AAG TCA AAG CTT TCA ACT	*‘Ca.* Annandia’ in *Adelges tsugae*
PinGam1_61f	TTG TCA TCT AAC TTA AAC AA	*‘Ca.* Annandia’ in *Pineus similis*
PinGam1_1161r	GAT TAA AAG TCT TGC TTC CAA CC	*‘Ca.* Annandia’ in *Pineus similis*
**Probes**		
b125	CAC TCT AAG ACA CGT TCC GA	*‘Ca.* Vallotia’ in *A. abietis, A. lariciatus*
b187	CCG CTT TCC TCC TTA GAG AAT	*‘Ca.* Vallotia’ in *A. cooleyi*
b442	TGC CAG GTT TTT TTC TTC TCG G	*‘Ca.* Vallotia’ in *A. cooleyi*
b1025	GTT AGT TCT CTT TCG AGC ACC	*‘Ca.* Vallotia’ in *A. cooleyi*
Al-b70	AGG CCG AAG CCT GCG TT	*‘Ca.* Vallotia’ in *A. laricis*
Al-b152	ATT CGG CTT TCG CCG GG	*‘Ca.* Vallotia’ in *A. laricis*
Al-b1256	CCC TCA CGG GTT GGC AA	*‘Ca.* Vallotia’ in *A. laricis*
b1027	CGA TTC TCT TTC GAG CAC	*‘Ca.* Vallotia’ in *A. laricis*
g69	AGA GCA AGC CCT TTT GTG TTA C	*‘Ca.* Gillettellia’ in *A. cooleyi*
g439	GTA CTT TAC TTT TCT TTC TCG CTG	*‘Ca.* Gillettellia’ in *A. cooleyi*
g1128	GAG TTC CCA CCT TTA TAT GCT G	*‘Ca.* Gillettellia’ in *A. cooleyi*
Al-g1023	AGA GCT CCC GAA GGC ACT	*‘Ca.* Profftia’ in *A. laricis*
Al-g1128	GAG TTC CCA CCA TTA CGT GCT G	*‘Ca.* Profftia’ in *A. laricis*
PinGam2-470	GAC GAT ATT AGC ATC AAC G	*‘Ca.* Hartigia’ in *P. similis*
PinGam2-828	CTC CTC AAG GAA ACA ACC TCC A	*‘Ca.* Hartigia’ in *P. similis*

### Phylogenetic Analyses

All unique, fully sequenced, 16*S* sequences were submitted to the BLAST feature of the NCBI webpage^1^ to confirm their similarity to previously identified symbionts of Adelgidae. Sequences were aligned with related adelgid symbionts and outgroups; phylogenetic analyses were performed with Bayesian inference with MrBayes 3.2.2 (Huelsenbeck and Ronquist, 2001) and maximum-likelihood with PhyML (Guindon and Gascuel, 2003) plugins in Geneious (version 6.1.8; Biomatters Ltd.). Bayesian analysis was run with the GTR + gamma model with four rate categories, three heated chains and one cold chain, chain length of 1 million, subsampling frequency of 500, and burn-in of 10%. Maximum-likelihood analysis was performed with the same model, with gamma distribution parameter estimated, and 500 bootstrap replicates.

### Fluorescent *In Situ* Hybridization and Confocal Microscopy

Localization of endosymbionts by fluorescent *in situ* hybridization (FISH) was performed on *Adelges abietis, A. cooleyi, A. lariciatus, A. laricis*, and *Pineus similis*, with methods following von Dohlen et al. (2013). Briefly, insects were fixed, embedded in tissue-freezing medium, and cryosectioned. Sections were post-fixed and hybridized overnight with fluorescently labeled probes. Visualization of hybridized probes was achieved with a Bio-Rad MRC1024 laser-scanning confocal microscope equipped with lasers of 488, 568, and 647 nm wavelengths. Specificity of hybridizations was confirmed with negative controls (no-probe, excess unlabeled probe, and RNase digestion). The general eubacterial probe 1507r was used as a positive control. Probes specific to each symbiont were designed from 16*S* sequences obtained from cloning (**Table 2**). Probes were used singly and in mixtures to increase hybridization signal. For *A. cooleyi*, ‘*Ca*. Vallotia cooleyia’ was detected using a “beta mix” containing b187, b442, and b1025; ‘*Ca*. Gillettellia cooleyia’ was detected using a “gamma mix” consisting of g69, g439, and g1128. Simultaneous localizations of both symbionts were achieved using a combination of the beta mix and the gamma mix. In *A. laricis*, ‘*Ca*. Vallotia tarda’ was localized using the single probe b1027. ‘*Ca*. Profftia tarda’ was localized using a gamma mix containing Al-g1023 and Al-g1128. Both symbionts were detected simultaneously with a combination of a beta mix containing Al-b70, Al-b152, and Al-b1256, and the gamma mix. Due to limited material of *A. lariciatus* and *A. abietis*, successful hybridizations were obtained only for the eubacterial probe 1507r and for ‘*Ca*. Vallotia,’ using b125. In *P. similis*, ‘*Ca*. Annandia pinicola’ was localized with GamC_440 (von Dohlen et al., 2013), ‘*Ca*. Hartigia pinicola’ was localized using a mix of PinGam2-470 and PinGam2-828, and simultaneous detection was performed with a combination of these probes.

### Electron Microscopy

Samples of *A. cooleyi* and *A. abietis* were processed at the Utah State University Electron Microscopy Facility (Logan, UT, United States). Abdomens of live insects were dissected into a solution of 2.5% paraformaldehyde, 4% glutaraldehyde, and 0.2M HEPES buffer. Fixation was performed using a microwave technique^2^, with modified fixation times: the primary fixation was microwave exposure for 3X 40 s, cooling to 10°C between exposures and replacing tissue in fresh fixative, then rinsing in 0.2M HEPES for 3X 1 min at room temperature (RT), then microwave exposure 2X with 1% osmium and potassium ferrocyanate in 0.2M HEPES for 4X 40 s, cooling to 10°C between exposures, with an intermediate water wash for 4X 1 min at RT. Subsequently, 1% thiocarbohydrizide (a chelator) was added to the sample for 10 min at RT to enhance cellular membranes. Samples were ethanol and acetone dehydrated, and embedded into SPURR’s epoxy. Ultrathin sections were cut with an ultramicrotome, stained with uranyl acetate and visualized on a transmission electron microscope (Zeiss, Leo, model 902; Thornwood).

Samples of *P. pini* were processed at the University of Utah Electron Microscopy Core Facility (Salt Lake City, UT, United States). Whole, live insects were fixed in a buffer of 1% glutaraldehyde, 2.5% paraformaldehyde, 100 mM cacodylate buffer (pH 7.4), 6 mM CaCl_2_, 4.8% sucrose for 3–4 days, after vacuum treatment to encourage submergence. Specimens were washed 3X for 15 min with cacodylate buffer, fixed with 2% osmium tetroxide at room temp for 1 h at room temperature (RT), washed 2X with cacodylate buffer and 1X with dH_2_O, then stained with saturated uranyl acetate for 1 h at RT, washed 3X with dH_2_O, and dehydrated with a graded ethanol series ending with absolute acetone, and infiltrated with a graded series of Epon epoxy resin (1:1 resin:acetone for 24 h, 3:1 resin:acetone for 24 h, 100% resin for 8 h with four changes). Samples were polymerized for 48 h at 60°C, ultrathin sectioned, and visualized on a JEOL JEM-1400 Plus transmission electron microscope.

## Results

### Symbiont Sequences and Adelgid-Symbiont Cospeciation

All adelgid species and populations yielded the expected two bacterial 16*S* sequences, which were highly similar to those of previously identified adelgid symbionts (**Table 3**). Identities of symbionts were consistent within adelgid species and within each alternate-conifer lineage. All populations of *A. cooleyi* from the Douglas-fir (*Pseudotsuga*) lineage yielded ‘*Ca*. Vallotia cooleyia’ and ‘*Ca*. Gillettellia cooleyia.’ All samples of *Adelges* from the larch (*Larix*) lineage (*A. abietis, A. japonicus, A. lariciatus, A. laricis*) yielded sequences of ‘Ca. Vallotia spp.’ and ‘Ca. Profftia spp.’ The Taiwan sample of *A. tsugae* from the hemlock (*Tsuga*) lineage yielded both ‘*Ca*. Annandia adelgestsuga’ and ‘*Ca*. Pseudomonas adelgestsugas.’ All samples of *Pineus* from the pine (*Pinus*) lineage yielded both ‘*Ca*. Annandia spp.’ and ‘*Ca*. Hartigia spp.’

**Table 3:** Characteristics of 16*S* rRNA genes of dual-obligate symbionts in Adelgidae.

Alternate-conifer host lineage and symbionts	Length (bp)	Ave. % identity within lineage	Ave. % GC	Related free-living bacteria	% GC from related free-living bacteria	Physical arrangement of symbionts
On Pine (*Pineus*)						
‘*Ca*. Annandia pinicola’	~1800^1^	97.7	44.8^2^/10.0^3^	*Erwinia billingiae*	55.2	Mostly in separate bacteriocytes; ‘*Ca*. Hartigia’ occupy central cells
‘*Ca*. Hartigia pinicola’	~1500	97.5	50.8	*Xenorhabdus bovienii*	54.0	
On Hemlock (*Adelges*)						
‘*Ca*. Annandia adelgestsuga’	~2100^1^	99.7	44.5^2^/11.3^3^	*Erwinia billingiae*	55.2	‘*Ca*. Annandia’ occupy bacteriocytes; ‘*Ca*. Pseudomonas’ occupy hemocoel
‘*Ca*. Pseudomonas adelgestsugas’	~1500	98.7	52.1	*Pseudomonas aeruginosa*	54.3	
On True Fir (*Adelges*)^4^						
‘*Ca*. Ecksteinia adelgidicola’	~1500	99.7	48.8	*Serratia proteamaculans*	54.2	Mostly in separate bacteriocytes; ‘*Ca*. Ecksteinia’ occupy central cells
‘*Ca*. Steffania adelgidicola’	~1500	99.6	51.8	*Pectobacterium carotovorum*	54.2	
On Douglas-fir (*Adelges*)						
‘*Ca*. Gillettellia cooleyia’	~1500	99.9	50.5	*Serratia proteamaculans*	54.2	Both symbionts intermixed in all bacteriocytes
‘*Ca*. Vallotia cooleyia’	~1500	99.8	53.2	*Burkholderia plantarii*	54.8	
On Larch (*Adelges*)						
‘*Ca*. Vallotia’ spp.	~1500	98.3	53.0	*Burkholderia plantarii*	54.8	Both symbionts intermixed in all bacteriocytes
‘*Ca*. Profftia’ spp.	~1500	97.1	50.8	*Obesumbacterium proteus*	53.7	

*All symbionts except ‘*Ca*. Vallotia spp.’ are Gammaproteobacteria; ‘*Ca*. Vallotia spp.’ are Betaproteobacteria.*

*^1^Extrapolated from alignment/comparison with *Buchnera* 16*S*.*

*^2^In 3′ alignable region.*

*^3^In extra 5′ region.*

*^4^Data from Toenshoff et al. (2012a).*

Average GC composition varied among symbionts (**Table 3**). Most were close to 50% or slightly higher, except for three below 50%: ‘*Ca*. Annandia adelgestsuga’ (44.5%), ‘*Ca*. A. pinicola’ (44.8%), and ‘*Ca*. Ecksteinia adelgidicola’ (48.8%). These averages deviated from the nearest free-living relatives by at least 1.6% and up to 45.2% in the most extremely degenerated regions.

The 5′ end of 16*S* rDNA from ‘*Ca*. Annandia spp.’ from *P. similis* and *A. tsugae* (sample NH04-36 from von Dohlen et al., 2013) was successfully amplified with the newly designed, specific primers. These 16*S* regions, when aligned with *E. coli* and closely related symbionts such as *Buchnera aphidicola* and *Purcelliella* strains, were longer, highly divergent, and highly AT-rich. The 5′ region contained approximately 380 extra base pairs (bp) in ‘*Ca*. Annandia pinicola’ from *P. similis* and approximately 750 extra bp in ‘*Ca*. Annandia adelgestsuga’ from *A. tsugae*, with G+C content of 8–10% (**Table 3**). This region was also unalignable between the two ‘*Ca*. Annandia’ species. Alignment of the ‘*Ca*. Annandia’ sequence from *Pineus similis* with the previously published ‘*Ca*. Annandia pinicola’ 16*S* sequence from *Pineus strobi* (Toenshoff et al., 2014; GenBank KC764418) revealed that this 1463 bp sequence is a chimera. While the approximately 1200 bp region from the 3′ end was 97% identical to the ‘*Ca*. Annandia’ sequence from *P. similis*, the approximately 230 bp region from the 5′ end was essentially unalignable with that sequence. BLAST searches in GenBank with this region returned 100% identity to Betaproteobacteria, principally, uncultured Burkholderiales and *Delftia* spp.

**FIGURE 1:**
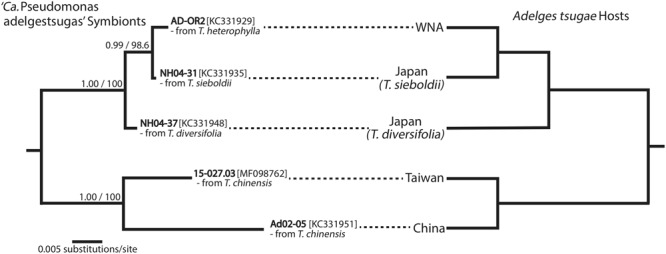
Cospeciation of *‘Ca.* Pseudomonas adelgestsugas’ symbionts and *Adelges tsugae* hosts from five genetically divergent populations [genetic divergence among *A. tsugae* suggests some populations may be distinct species (Havill et al., 2006, 2016)]. Phylogeny of *‘Ca.* Pseudomonas adelgestsugas’ symbionts (left) was estimated from 16*S* rRNA sequences. *A. tsugae* phylogeny (right) was simplified from Havill et al. (2006) and Havill et al. (2016), in which all population-level nodes were significantly supported. Sample from Taiwan is new to this study. WNA, western North America.

Assessment of cospeciation between symbionts and adelgid hosts was partially inconclusive. Relationships of adelgid symbionts within each symbiont lineage were not universally resolved with confidence, with one exception (**Figure 1** and Supplementary Figures 1–4). Most symbiont phylogenies generated from 16*S* sequences yielded low support at several internal nodes. This, combined with low confidence in some nodes of *Adelges* host relationships (Havill et al., 2007), precluded definitive tests of cospeciation in most cases. However, comparison of ‘*Ca*. Vallotia’ with *Adelges* topologies suggested nearly congruent relationships (Supplementary Figure 1). Furthermore, a strict cospeciation pattern was recovered between ‘*Ca*. Pseudomonas adelgestsugas’ and *Adelges tsugae* host populations (**Figure 1**).

### Localization and Ultrastructure of Endosymbionts

**FIGURE 2:**
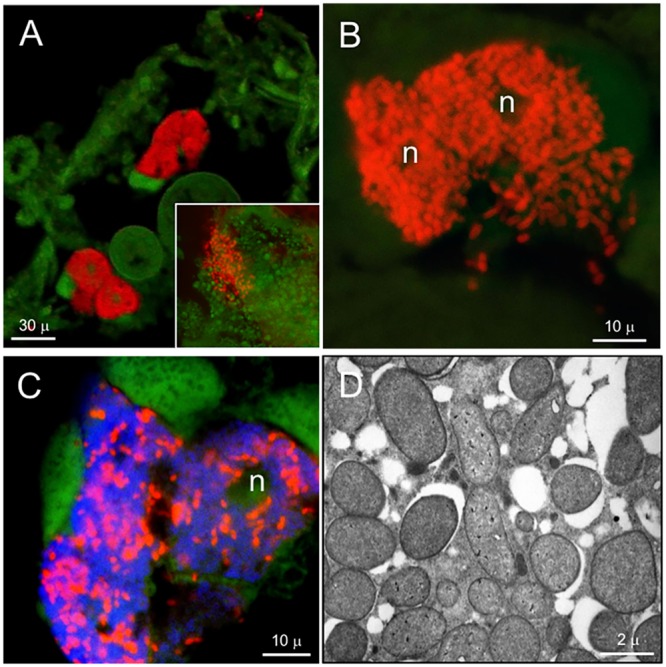
Localization and ultrastructure of bacteriocyte-associated endosymbionts in *A. cooleyi*. **(A)** General detection of symbionts in paired bacteriomes of second-instar stage from spruce galls (transverse section) by fluorescence *in situ* hybridization (FISH), using general eubacterial probe 1507r labeled with Alexa-568-5-dUTP (red). Inset: general detection of symbionts, as above, in an egg. **(B)** Detection of ‘*Ca*. Vallotia cooleyia’ in second-instar stage from spruce galls (transverse section) by FISH, using the beta probe mix of b187, b442, and b1025 labeled with Alexa-568-5-dUTP (red). **(C)** Simultaneous detection of both symbionts in second-instar stage from spruce galls (transverse section) by FISH, using the beta mix (as above) labeled with Bodipy-650-14-dUTP for ‘*Ca*. Vallotia cooleyia’ (blue), and gamma probe mix of G-69, G-439, and G-1128 labeled with Alexa-568-5-dUTP for ‘*Ca*. Gillettellia cooleyia’ (red). **(D)** TEM micrograph of bacteriocyte containing two bacterial morphotypes. Green, autofluorescence of insect cuticle; n, bacteriocyte nucleus.

Results of microscopy showed that, for all geographic and host-conifer populations of each adelgid species, the dual symbionts were consistently located within host bacteriocyte cells, and in two basic physical arrangements (**Table 3**). FISH performed with the general eubacterial probe on *Adelges cooleyi* nymphs from spruce galls revealed paired bacteriomes densely filled with bacteria (**Figure 2A**). Hybridizations with the betaproteobacterial probe mix localized ‘*Ca*. Vallotia cooleyia’ to the bacteriocytes and indicated that this symbiont was numerous within host cells (**Figure 2B**). Hybridizations with the gammaproteobacterial probe mix localized ‘*Ca*. Gillettellia cooleyia’ to bacteriocytes, but suggested that these symbionts were less numerous in host cells of some insects at this stage. Transverse sections hybridized simultaneously with beta- and gammaproteobacterial probes confirmed that bacteriocytes harbored both symbiont species, which were co-mingled within host cells (**Figure 2C**). In some hybridizations, certain bacteriocytes appeared to harbor mostly one symbiont species or the other; however, this phenomenon needs to be explored more rigorously with additional studies. Hybridizations with the general eubacterial probe in adult insects showed bacteria clustered at one pole of developing eggs; these were presumed to be vertically transferred endosymbionts (**Figure 2A** inset).

Transmission electron microscopy of *A. cooleyi* bacteriomes corroborated results of FISH. TEM micrographs showed numerous bacteria residing in bacteriocytes, with two different bacterial forms co-existing and intermixed within the same host cells. Both forms were of similar size and coccoid shape but one form stained darker than the other (**Figure 2D**).

**FIGURE 3:**
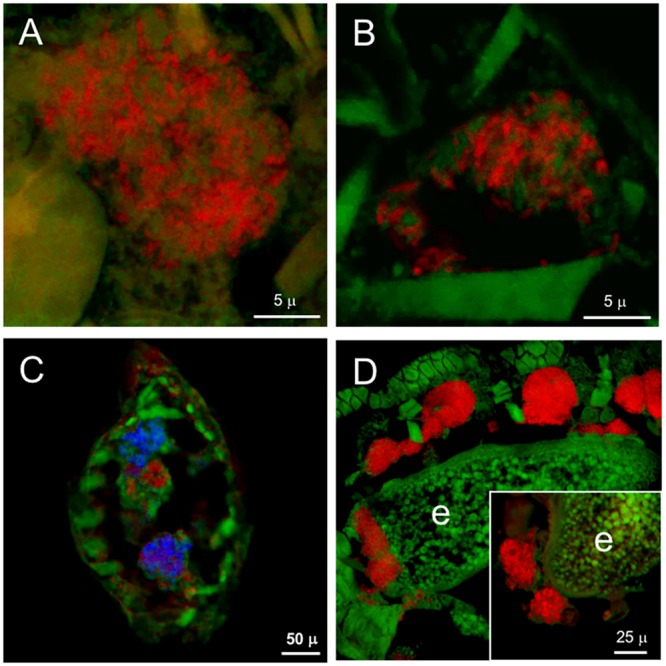
Localization of bacteriocyte-associated endosymbionts in *A. laricis* generations from larch by FISH. **(A)** Detection of ‘*Ca*. Vallotia tarda’ in first-instar stage from larch generations, using specific probe b1027 labeled with Alexa-568-5-dUTP (red). **(B)** Detection of ‘*Ca*. Profftia tarda’ in first-instar stage from larch generations, using specific probe g1023 labeled with Alexa-568-5-dUTP (red). **(C)** Detection of both symbionts in paired bacteriomes of first-instar stage from larch generations (transverse section), using beta mix Al-b70 + Al-b152 + Al-b1256 labeled with Alexa-568-5-dUTP for ‘*Ca*. Vallotia tarda’ (red), and gamma mix Al-g1023 + Al-g1128 labeled with Bodipy-650-14-dUTP for ‘*Ca*. Profftia tarda’ (blue). **(D)** Adult generation with eggs, probed with general eubacterial probe 1507r labeled with Alexa-568-5-dUTP (red); individual bacteriocytes surround the egg, which contains a cluster of transmitted symbionts at the posterior pole (left). Inset: ‘*Ca*. Vallotia tarda’ clustered at the posterior pole of an egg, detected with specific probe b1027 labeled with Alexa-568-5-dUTP (red). Green, autofluorescence from insect cuticle; e, egg.

Fluorescent *in situ* hybridization performed on *Adelges laricis* from larch with either gamma- or betaproteobacterial-specific probes in first-instar/crawler stages indicated numerous cells of both ‘*Ca*. Vallotia tarda’ and ‘*Ca*. Profftia tarda’ within the bacteriome. The bacteriome appeared to be in a syncytial phase at this early stage of development (**Figures 3A,B**). First-instars hybridized simultaneously with beta- and gammaproteobacterial probes suggested that some bacteriocytes—or areas of the bacteriome—may harbor mostly one symbiont or the other, while other regions appear to contain a mixture of both bacterial types (**Figure 3C**). By the fourth-instar/adult stage, both symbionts were intermixed within well-defined bacteriocytes (Supplementary Figure 5). Individual hybridizations with the general eubacterial and betaproteobacterial probes demonstrated vertical transmission of endosymbionts: bacteriocytes containing symbionts were observed adjacent to oocytes and ‘*Ca*. Vallotia tarda’ cells were detected within oocytes (**Figure 3D**). While we infer that both endosymbiont species are transferred vertically in these populations (due to their presence in pre-feeding crawlers), we did not directly confirm the vertical transmission of ‘*Ca*. Profftia tarda.’

Successful FISH images for *Adelges abietis* and *A. lariciatus* from spruce galls were obtained only with the general eubacterial probe and ‘*Ca*. Vallotia’-specific probes. All results localized symbionts to the bacteriome. Specific FISH for ‘*Ca*. Vallotia’ indicated that this symbiont was found in all bacteriocytes, and was co-mingled with other bacteria that were presumed to be ‘*Ca*. Profftia’ (Supplementary Figure 6). TEM of *A. abietis* bacteriocytes revealed an arrangement similar to *A. cooleyi*. Bacteriocytes were packed with bacterial cells, but showed two distinct shapes and staining patterns, one lighter-staining and polymorphic and one darker-staining and coccoid. As in *A. cooleyi*, both symbiont forms in *A. abietis* were similar in size (Supplementary Figure 7).

**FIGURE 4:**
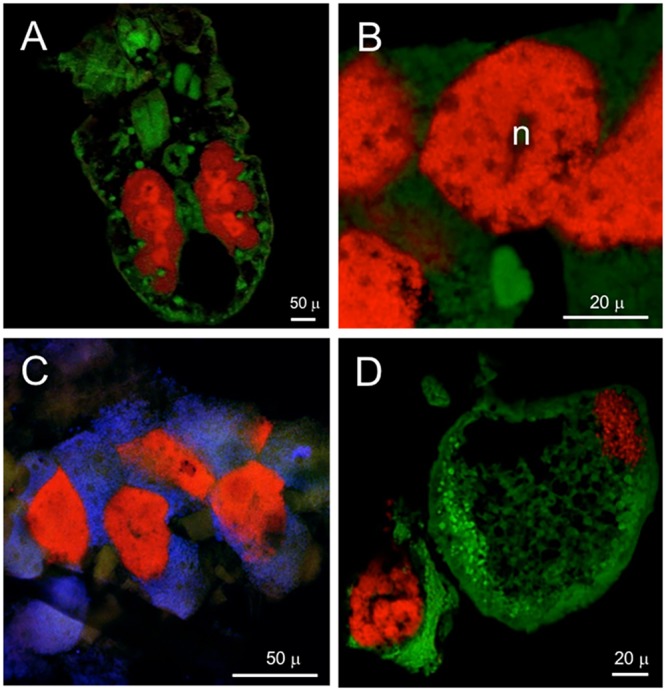
Localization of bacteriocyte-associated endosymbionts in *P. similis* by FISH. **(A)** General detection of both symbionts of second-instar stage (frontal section) using eubacterial probe 1507r labeled with Alexa-568-5-dUTP (red). Central bacteriocytes containing ‘*Ca*. Hartigia pinicola’ fluoresce more brightly with this probe. **(B)** Detection of ‘*Ca*. Hartigia pinicola’ symbionts within central bacteriocytes of a second-instar stage using a mix of PinGam2-470 and PinGam2-828 labeled with Alexa-568-5-dUTP (red). **(C)** Simultaneous detection of ‘*Ca.* Annandia pinicola’ using GamC-440 labeled with Bodipy-650-14-dUTP (blue), and ‘*Ca*. Hartigia pinicola’ using PinGam2-470 and PinGam2-828 labeled with Alexa-568-5-dUTP (red). **(D)** General detection of both symbionts in an egg using eubacterial probe 1507r labeled with Alexa-568-5-dUTP (red). A cluster of symbionts is located at the posterior pole (upper right); other symbionts from the disintegrated bacteriome are clustered on the lower left. Green, autofluorescence of insect cuticle; n, bacteriocyte nucleus.

**FIGURE 5:**
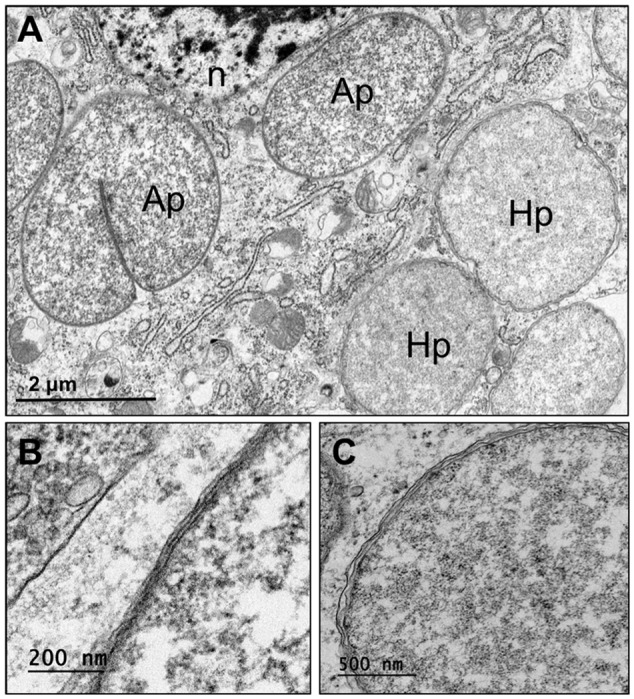
Ultrastructure of *Pineus pini* endosymbionts residing in bacteriocytes. **(A)** Ultrathin section of bacteriome in whole-mount insects, showing two distinct bacteriocytes containing ‘*Ca*. Annandia pinicola’ (left) and ‘*Ca*. Hartigia pinicola’ (right). **(B)** High magnification of ‘*Ca*. Annandia pinicola’ cell envelope, comprising three membrane layers, presumably corresponding to inner and outer membranes and a symbiosome membrane. **(C)** High magnification of ‘*Ca*. Hartigia pinicola’ cell envelope, comprising three membrane layers, presumably corresponding to inner and outer membranes and a symbiosome membrane; no peptidoglycan layer is apparent. Ap, ‘*Ca*. Annandia pinicola’; Hp, ‘*Ca*. Hartigia pinicola’; n, bacteriocyte nucleus.

Frontal sections of second-instar *P. similis* from spruce galls hybridized with the general probe 1507r revealed a large, paired bacteriome packed with symbionts (**Figure 4A**). More intense fluorescence was observed in the centrally located bacteriocytes. FISH with ‘*Ca*. Hartigia pinicola’-specific probes indicated that these symbionts occupied the central bacteriocytes exclusively (**Figure 4B**). Co-labeling with probes specific to each symbiont confirmed the former result and localized ‘*Ca*. Annandia pinicola’ exclusively to peripheral bacteriocytes within the bacteriome (**Figure 4C**). Fluorescent signal from ‘*Ca*. Annandia’ was much weaker than that from ‘*Ca*. Hartigia’ (**Figures 4A,C**). FISH with egg-bearing adults and the general eubacterial probe demonstrated vertical transmission of bacteria to eggs (**Figure 4D**). Additional experiments with specific probes confirmed that these cells represent both symbiont species (Supplementary Figure 8). Ultrastructure of endosymbionts from *P. pini* adults from pine showed two distinct forms occupying different bacteriocytes: the slightly darker-staining and pleomorphic ‘*Ca*. Annandia pinicola’ in outer bacteriocytes and the slightly lighter-staining coccoid ‘*Ca*. Hartigia pinicola’ in central bacteriocytes (**Figure 5**). Cell envelopes of both symbionts exhibited three layers, which were presumed to represent inner and outer membranes and the symbiosome membrane. Unlike symbionts of *P. strobi* (Toenshoff et al., 2014), no peptidoglycan layer was apparent in ‘*Ca*. Hartigia pinicola’ cell walls, nor were vesicles visible between ‘*Ca*. Hartigia pinicola’ bacterial and symbiosome membranes.

### Proposed Taxonomy of New Symbionts

We propose names for the newly characterized symbionts, according to the recommendations of Murray and Stackebrandt (1995) and considering the phylogenetic affiliations and genetic distances to symbionts already described. To simplify the naming system, and be consistent with previous designations, we propose to retain the previously designated ‘*Ca*. Annandia pinicola’ and ‘*Ca*. Hartigia pinicola’ for symbionts of all *Pineus* spp. (herein, *P. coloradensis, P. similis*, and *P. pini*). Because two members of the larch-feeding (*Larix* spp.) lineage have already been given species-specific symbiont names (Toenshoff et al., 2012a), we propose to retain this system for the newly sampled species. For the betaproteobacterial symbionts of *A. japonicus* and *A. lariciatus* feeding on alternate-host larch, we propose *‘Candidatus* Vallotia japonica’ and *‘Candidatus* Vallotia lariciata.’ For the gammaproteobacterial symbionts from *A. japonicus* and *A. lariciatus*, we propose *‘Candidatus* Profftia japonica’ and *‘Candidatus* Profftia lariciata.’

We also propose new terminology to differentiate co-symbionts according to their hypothesized seniority and obligate status in the association. The historical usage of “primary” and “secondary” symbionts has been applied to both obligate and facultative associates. Furthermore, these labels are potentially confusing when discussing insect hosts with complex life cycles that also use the terminology of primary and secondary host plants. To alleviate these issues we propose the use of “senior symbiont” and “junior symbiont” for obligate co-symbionts of older and younger associations, respectively. We employ these terms for the remainder of the paper.

## Discussion

### Dual-Obligate Symbionts of Adelgids and a Model for Lineage-Specific Replacements

Previous work on adelgid endosymbionts uncovered a surprising diversity of bacterial lineages; in addition, these studies suggested that all adelgid species harbor two endosymbionts residing in the bacteriome (Toenshoff et al., 2012a,b, 2014; Michalik et al., 2013; von Dohlen et al., 2013), with one exception: in *Adelges tsugae*, one symbiont is housed in the hemocoel (von Dohlen et al., 2013). While this previous work implied that the dual bacteria may be obligate partners, some uncertainty remained. Namely, for some adelgid species only one population was sampled, or samples came from only one host plant or geographic area. In addition, in *Adelges piceae*, a genome fragment from ‘*Ca*. Steffania adelgidicola’ appeared to be characteristic of a facultative (non-obligate) symbiont (Toenshoff et al., 2012b). In *Adelges tsugae*, the hemocoel-residing symbiont (‘*Ca*. Pseudomonas adelgestsugas’) was not recovered from one population (von Dohlen et al., 2013). Furthermore, the symbiont of *A. tsugae* that populated central bacteriocytes was highly similar in 16*S* sequence to a known facultative symbiont of aphids, *‘Ca*. Serratia symbiotica’ cluster A; (Burke et al., 2009). Moreover, this symbiont was found in only one of five native populations (von Dohlen et al., 2013). Thus, questions remained regarding the ubiquity of dual symbionts within Adelgidae and the nature of their relationships with host insects.

The present study sought to resolve whether bacteria found in adelgids are obligate symbionts, co-obligate symbionts, facultative symbionts, or a mixture of these. This information bears on questions concerning which symbionts represent ancient associations and how symbionts may have been gained, lost, and/or replaced over adelgid history. We expect that long-term, obligate partners should be present in all populations of every host species, be vertically transmitted, and show cospeciation with host insects (Munson et al., 1991; Chen et al., 1999). We further expect to find other characteristics of long-term symbiosis, for example, nucleotide compositional bias (Lind and Andersson, 2008; McCutcheon and Moran, 2012) and circular or pleomorphic cell shape indicative of the loss of cell-envelope biosynthesis genes (McCutcheon and Moran, 2010; McCutcheon and von Dohlen, 2011).

Here, we found that previously identified pairs of bacteriome residents indeed showed characteristics of long-term mutualists. All pairs of symbionts were recovered from all samples and species, as expected. Where assayed with FISH, both symbionts were found in the bacteriome, with the exception of *A. tsugae*, as noted above (von Dohlen et al., 2013). *‘Ca*. Pseudomonas adelgestsugas’ is determined to be obligate because it was successfully recovered in this study from the Taiwan population, where previous work had failed to detect it (likely due to limited, low-quality material) (von Dohlen et al., 2013). Evidence for vertical transmission was also observed in the new samples and species assayed here, as in previous studies.

Strict or nearly strict cospeciation of endosymbionts and insect hosts was inferred for some endosymbionts, but not all. Only for *‘Ca*. Pseudomonas,’ the hemocoel symbiont of *A. tsugae*, could we confirm strict cospeciation with confidence (**Figure 1**). Cospeciation was nearly perfect for *‘Ca*. Vallotia’ symbionts and their hosts, but was less evident for other symbionts, which showed various levels of incongruence. In most comparisons, either symbiont phylogeny, host phylogeny, or both, contained some level of uncertainty. Thus, lack of cospeciation patterns could be a consequence of low information content in sequences. Additional data will be needed to resolve relationships of both symbionts and adelgid hosts with full confidence.

The *‘Ca*. Serratia symbiotica’ symbiont detected previously in one native Japanese population of *A. tsugae* was previously presumed to be a facultative symbiont (von Dohlen et al., 2013), similar to *S. symbiotica* found in several aphids and *Bemisia tabaci* whiteflies. This symbiont was not detected previously in any other adelgid species (Toenshoff et al., 2012a,b, 2014), nor was it found in any of the new species or samples assayed here. Wider sampling of adelgid species and populations could determine whether this facultative symbiont is limited to *A. tsugae*, only.

**FIGURE 6:**
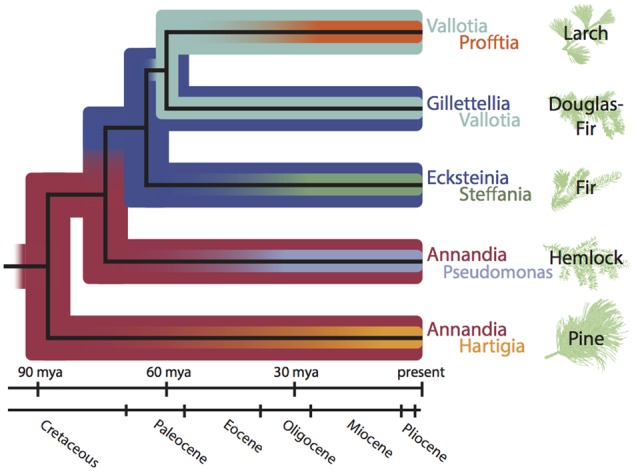
Hypothesis of symbiont acquisitions and replacements during Adelgidae evolutionary history (dated phylogram based on Havill et al., 2007). This scenario posits ‘*Ca*. Annandia’ as the original, ancestral “senior” symbiont. ‘*Ca*. Annandia’ was joined by the “junior” symbiont ‘*Ca*. Hartigia’ in the *Pineus* lineage (feeding on alternate-host pine), and by the junior symbiont ‘*Ca*. Pseudomonas’ in the *A. tsugae* species complex (on alternate-host hemlock). ‘*Ca*. Annandia’ was replaced by the *Serratia*-type ancestor of ‘*Ca*. Ecksteinia’ and ‘*Ca*. Gillettellia’ (these symbionts are sister taxa within the gammaproteobacterial *Serratia* lineage) to become the new senior symbiont in the fir + Douglas-fir + larch lineage. ‘*Ca*. Ecksteinia’ was joined by junior symbiont ‘*Ca*. Steffania’ in the fir lineage, and ‘*Ca*. Gillettellia’ was joined by junior symbiont ‘*Ca*. Vallotia’ in the ancestor of the Douglas-fir + larch lineage. In the ancestor of the larch lineage, the *Serratia*-type symbiont was lost; ‘*Ca*. Vallotia’ remained to become the new senior symbiont, and was joined by junior symbiont ‘*Ca*. Profftia.’

We propose a scenario of gains, losses, and replacements to explain the pattern of co-obligate symbiont diversity in Adelgidae (**Figure 6**). Under this model, within each adelgid lineage one symbiont is older (“senior symbiont”) and one is younger (“junior symbiont”). *‘Ca*. Annandia’ is assumed to be the ancestral, senior symbiont of adelgids because it is shared by both *Pineus* and the basal *Adelges* lineage, *A*. *tsugae*; this designation is further supported by its highly pleomorphic cell shape (von Dohlen et al., 2013) and anomalous, GC-poor 16*S* rRNA 5’ insertion sequence. This senior symbiont was replaced twice successively, and different junior symbionts were gained in five separate events. Notably, our hypothesis suggests that *‘Ca*. Vallotia’ changed status from a junior symbiont in the ancestor of the Douglas-fir + larch lineage to a senior symbiont in the ancestor of the larch lineage. Notably, many of the acquisition and replacement events are coincident with acquisitions of different alternate-conifer hosts in each major adelgid lineage (**Figure 6**), which may have occurred in the early Paleogene (Havill et al., 2007).

Most lineages of sap-feeding insects have experienced novel acquisitions, replacements, and losses of obligate symbionts. In Auchenorrhynchans (cicadas and various hoppers), several junior symbionts have joined the ancient *Sulcia* symbiont in a dual-obligate role, and have also been replaced (Bennett and Moran, 2013). The senior *Carsonella* symbiont of psyllids and the *Tremblaya* symbiont of mealybugs have each been joined by co-symbionts (Thao et al., 2000a, 2002; Spaulding and von Dohlen, 2001; Hall et al., 2016; Husnik and McCutcheon, 2016). While aphids almost universally and exclusively harbor *Buchnera* as the sole nutritional symbiont, some *Cinara* aphids acquired *Serratia* bacteria as an obligate, nutritional junior symbiont (Lamelas et al., 2011; Manzano-Marin et al., 2016). In planthoppers (Fulgoroidea), *Sulcia* may have been lost in certain subfamilies (Moran et al., 2005; Urban and Cryan, 2012). Clearly, obligate symbionts may come and go, but in general, the ancient senior symbiont is most often retained in descendant host-insect species, while replacements and/or novel acquisitions occur mostly for junior symbionts. Adelgids, therefore, present an unusual case–in a comparatively young lineage–of multiple replacements of both the senior symbiont and the more recent junior symbionts.

Similar to the obligate, maternally transmitted symbionts of other sap-feeding insects, we presume that the five pairs of dual-obligate symbionts in Adelgidae are nutritional partners of their hosts. As in several other systems (Wu et al., 2006; McCutcheon and Moran, 2007, 2010; McCutcheon and von Dohlen, 2011), dual symbionts of adelgids are likely to be cooperating with each other and with the host insect to accomplish nutrient synthesis. We are currently sequencing genomes to characterize symbiont and host roles in this capacity; preliminary data indeed support a nutritional role for symbionts in this system (Weglarz et al., in preparation).

In nutritional symbioses, acquisitions of junior symbionts are thought to be compensatory events that rescue the consortium from potentially debilitating mutations within symbiont genomes (Bennett and Moran, 2015). Due to their unusual population structure compared to free-living bacteria, symbionts may accumulate deleterious mutations through the effects of strong genetic drift (Moran, 1996). Such mutations can involve loss of key enzymes within nutrient-synthesis pathways (Rispe and Moran, 2000; Wernegreen, 2002). By acquiring novel, junior symbionts with complete pathways, host insects can restore biosynthetic capabilities (McCutcheon et al., 2009; McCutcheon and Moran, 2010; Sloan and Moran, 2012). However, once sequestered within hosts, junior symbionts become captive to the same mutational processes (Bennett and Moran, 2015).

Most obligate senior symbionts of hemipterans have persisted since the origin of their host-insect lineages in the Jurassic-Cretaceous Periods, and many junior symbiont associations are also old (Munson et al., 1991; Spaulding and von Dohlen, 1998; Moran et al., 2005; Kuechler et al., 2013). Complete replacement of a lineage’s senior symbiont appears to be rare. As such, host-level selection must be acting to preserve the original symbiont despite strong genetic drift. Where ancient (senior or junior) symbionts have been lost or replaced, a change in host-level selection can be presumed. For example, Typhlocybinae leafhoppers appear to have lost both the senior *Sulcia* and junior *Nasuia* co-symbionts, presumably as a consequence of shifting from a diet of phloem to nutrient-rich parenchyma (Buchner, 1965; Bennett and Moran, 2015). Phylloxerans (closest relatives of aphids and adelgids) have apparently lost symbionts entirely on a diet of nutrient-rich parenchyma in galls (Buchner, 1965; Warick and Hildebrandt, 1966; Nabity et al., 2013). Losses and replacements of symbionts in adelgids might be similarly tied to historical changes in nutritional resources.

### Hypothesis of Historical Fluctuations in Nutritional Demands to Explain Symbiont Dynamics

Adelgids are unusual among sap-feeding insects with respect to their feeding mode. While most sap-feeding insects exploit a single plant tissue (i.e., phloem, xylem, or parenchyma), adelgids utilize both phloem and parenchyma over the course of their multi-generational life cycles, in a generation-specific manner. In host-alternating species, generations on alternate-conifer needles tap phloem, but generations on spruce branches and inside galls tap parenchyma cells (Balch and Underwood, 1950; Auclair, 1963; Pollard, 1973). Exceptions may occur in species with simplified life cycles (Annand, 1928; Blackman and Eastop, 1994; Young et al., 1995). Parenchyma storage cells provide a high-nitrogen, near-complete diet (Cowling and Merrill, 1966; Hillis, 1987), but conifer phloem has a typical low-nitrogen profile (Näsholm et al., 1987; Schneider et al., 1996; Gessler et al., 1998). Thus, many extant adelgids alternate between phases of high nutrition and phases of low nutrition over the course of their life cycles. Consequently, host-alternating adelgids should experience weak selection for symbiont nutritional functions in parenchyma-feeding generations and strong selection for nutritional functions in phloem-feeding generations of the life cycle.

We propose that the unusual degree of symbiont turnover in adelgids may reflect historical fluctuations in selection for nutritional provisioning by symbionts, consequent with acquisitions of new alternate-conifer hosts and evolution of their host-alternating life cycles. We hypothesize the following sequence: the adelgid common ancestor fed on spruce phloem and was supplemented by a single, ancestral nutritional symbiont. Parenchyma feeding and galls evolved later, relaxing selection for nutrient production in the symbiont and resulting in severe genome degradation. Chance acquisition of a junior co-symbiont restored full nutrient synthesis, and facilitated re-feeding on nitrogen-poor phloem. This allowed capture of an alternate conifer, thereby evolving host alternation. Capture of alternate conifers would be facilitated by phloem feeding, allowing adelgid migrants exploiting a novel conifer to bypass many plant defenses (Walling, 2008) using this feeding mode.

A logical deduction from this scenario is that independent origins of host alternation in adelgids have occurred in each of their five alternate-conifer lineages. This is because the hypothesis depends on prolonged evolutionary periods of parenchyma feeding on spruce, e.g., along the “backbone” of the adelgid phylogeny (**Figure 6**). Theory on the evolution of complex life cycles and host alternation includes elements of both adaptation and constraint (Moran, 1988, 1994; Mackenzie and Dixon, 1990). To evolve from simple life cycles, complex life cycles presumably have some adaptive advantage. Havill and Foottit (2007) proposed an adaptive hypothesis for host alternation in adelgids that incorporates the idea of cyclical escape from conifer defenses. It is reasonable to hypothesize that a highly adaptive strategy might have multiple origins within Adelgidae. The same has been proposed for host-alternating aphids (von Dohlen and Moran, 2000; Jousselin et al., 2010; Hardy et al., 2015).

## Summary

We have extended previous work on symbionts of adelgids to provide evidence that the bacterial pairs found in these insects are likely dual-obligate symbionts. We formulated the novel insight that specific pairs of symbionts correlate with, and are consistent within, major lineages of adelgids, which are in turn associated with unique alternate-conifer genera. We determined that the previously published 16*S* sequence of ‘*Ca*. Annandia pinifoliae’ (Toenshoff et al., 2014) is chimeric, and that the true 16*S* rRNA genes of ‘*Ca*. Annandia’ spp. contain lengthy and highly AT-rich expansions in the 5′ end. These unusual expansions support the assumption of ‘*Ca*. Annandia’ as the senior symbiont of adelgids. We also contend that the junior symbiont of *A. tsugae* is ‘*Ca*. Pseudomonas adelgestsugas,’ and not *Serratia symbiotica* as portrayed previously (Toenshoff et al., 2014). We propose a hypothesis detailing specific events in the dynamic acquisitions and losses of senior and junior symbionts in adelgids, which correlate with acquisitions of new alternate-conifer hosts. This hypothesis incorporates a transition in one symbiont lineage from junior to senior symbiont status. We speculate that this dynamism implies a complex history of interplay between symbiont turnover and life cycle evolution in Adelgidae.

## Author Contributions

CvD, KP, US, and KW conceived the research. KP, US, and KW performed the experiments. CvD, KP, US, and KW analyzed the data. CvD, KP, US, and GB wrote the manuscript. RF and NH contributed samples. All authors reviewed, edited, and accepted the manuscript.

## Conflict of Interest Statement

Author US is currently employed by BioFire Diagnostics, LLC.

All other authors declare that the research was conducted in the absence of any commercial or financial relationships that could be construed as a potential conflict of interest.

## Footnotes

1 http://www.ncbi.nlm.nih.gov

2 http://www.tedpella.com/Micro_html/vacpro.htm
