# Supplementary material for: Dynamic Acquisition and Loss of Dual-Obligate Symbionts in the Plant-Sap-Feeding Adelgidae (Hemiptera: Sternorrhyncha: Aphidoidea)
Source: Front Microbiol. 2017 Jun 13;8:1037. doi: 10.3389/fmicb.2017.01037 (PMC5468457; doi:10.3389/fmicb.2017.01037)
Supplement: Supplementary file 1 [file Presentation_1.PDF]

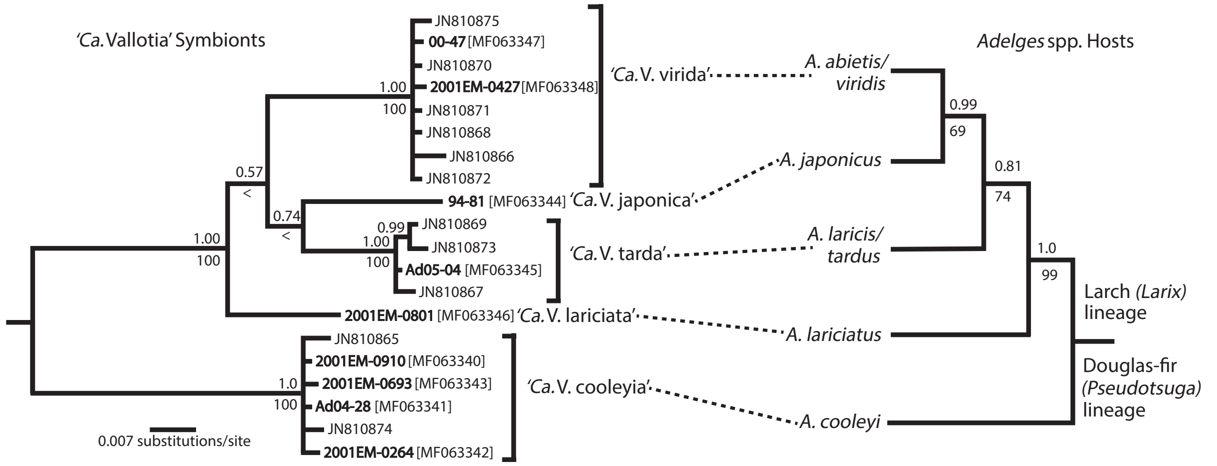

Supplementary Figure 1. Phylogeny of '*Ca*. Vallotia' symbionts estimated from 16S rRNA sequences (left) from five adelgid species. Adelgid phylogeny (right) was simplified from Havill *et al*. 2007. Numbers above and below branches are Bayesian posterior probabilities (BPP) and maximum-likelihood bootstrap percentages (MBP), respectively; "<" indicates BPP below 0.5 or MBP below 50%.

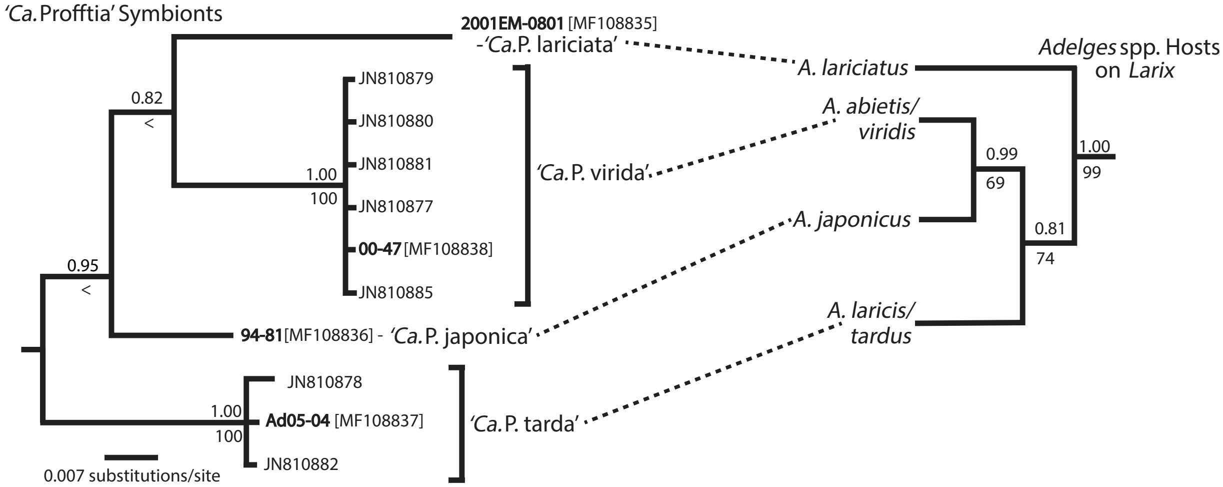

Supplementary Figure 2. Phylogeny of '*Ca*. Profftia' symbionts estimated from 16S rRNA sequences (left) from four adelgid species. Adelgid phylogeny (right) was simplified from Havill *et al*. 2007. Numbers above and below branches are Bayesian posterior probabilities (BPP) and maximum-likelihood bootstrap percentages (MBP), respectively; "<" indicates BPP below 0.5 or MBP below 50%.

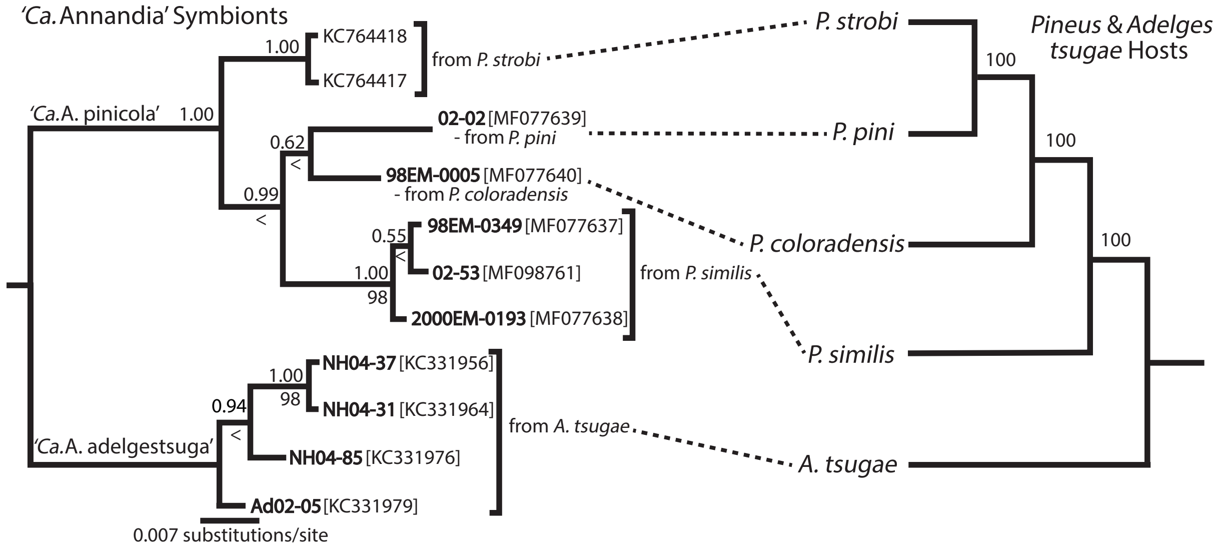

Supplementary Figure 3. Phylogeny of '*Ca*. Annandia' symbionts estimated from 16S rRNA sequences (left) from five adelgid species. Adelgid phylogeny (right) was simplified from Havill *et al*. 2007. Numbers above and below branches are Bayesian posterior probabilities (BPP) and maximum-likelihood bootstrap percentages (MBP), respectively; "<" indicates BPP below 0.5 or MBP below 50%.

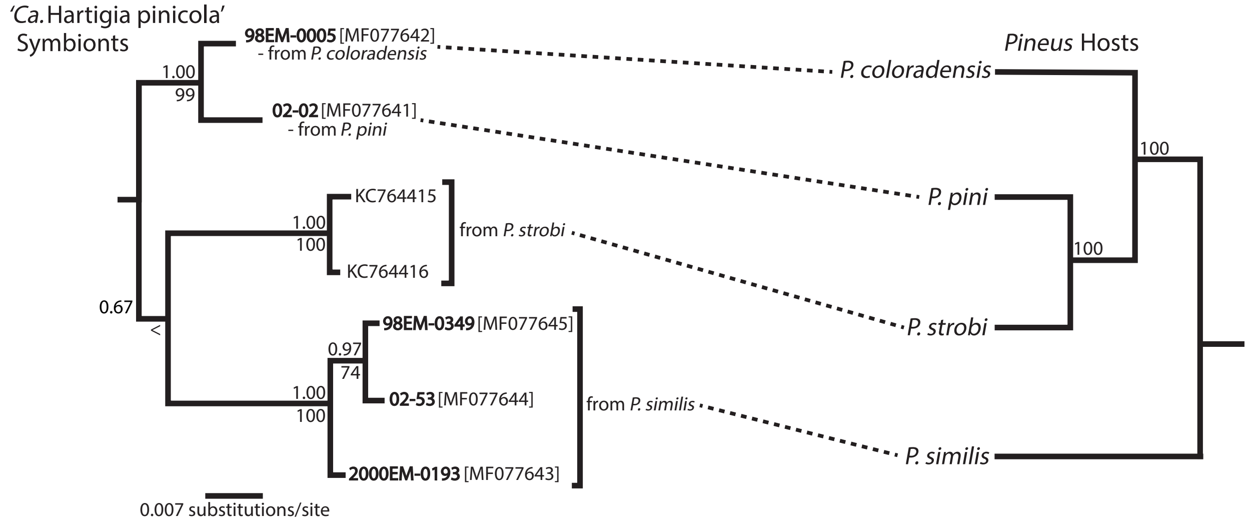

Supplementary Figure 4. Phylogeny of '*Ca*. Hartigia' symbionts estimated from 16S rRNA sequences (left) from four adelgid species. Adelgid phylogeny (right) was simplified from Havill *et al*. 2007. Numbers above and below branches are Bayesian posterior probabilities (BPP) and maximum-likelihood bootstrap percentages (MBP), respectively; "<" indicates BPP below 0.5 or MBP below 50%.

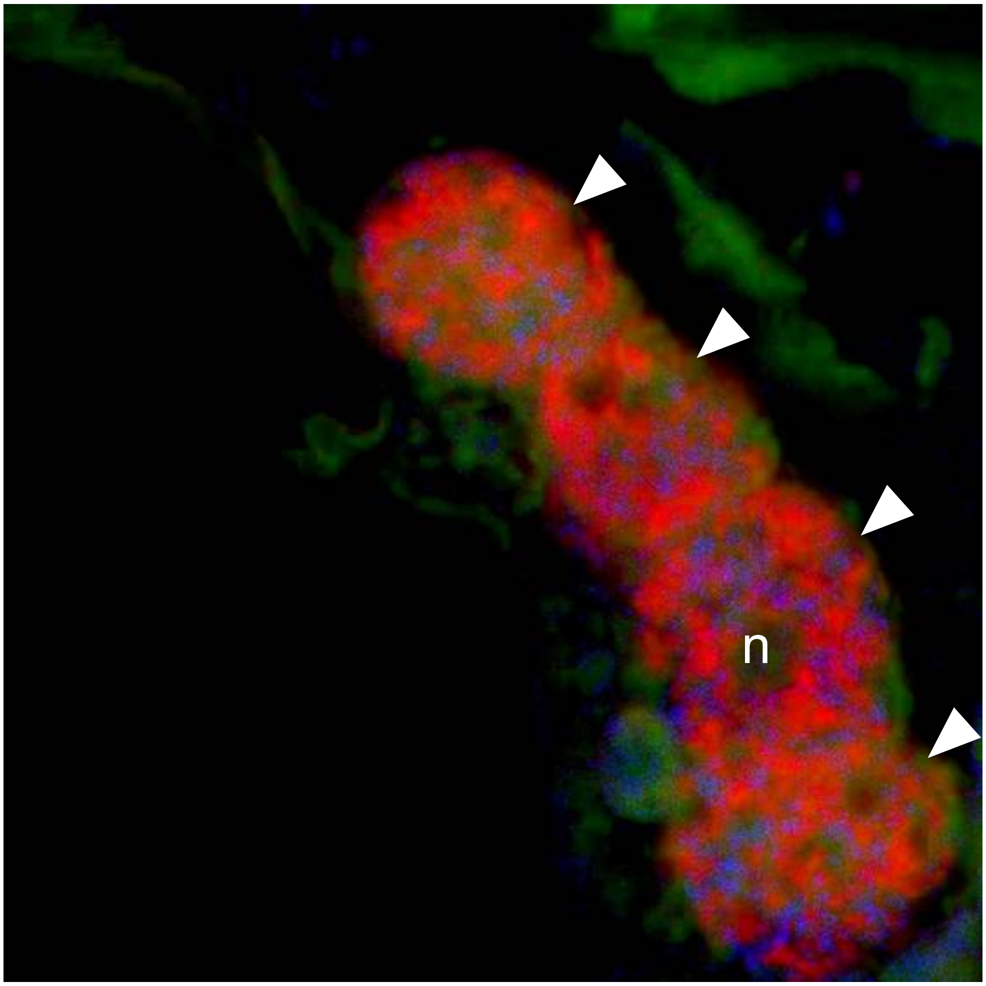

Supplementary Figure 5. Localization of bacteriocyte-associated endosymbionts of *Adelges laricis* by FISH. Detection of both symbionts in the bacteriomes of 4th-instar stage from larch generations (frontal section), using beta mix Al-b70 + Al-b152 + Al-b1256 labeled with Bodipy-650-14-dUTP for '*Ca*. Vallotia tarda' (blue), and gamma mix Al-g1023 + Al-g1128 labeled with Alexa-568-5-dUTP for '*Ca*. Profftia tarda' (red). White arrowheads point to individual bacteriocytes. Green, autofluorescence of insect cuticle; n, bacteriocyte nucleus.

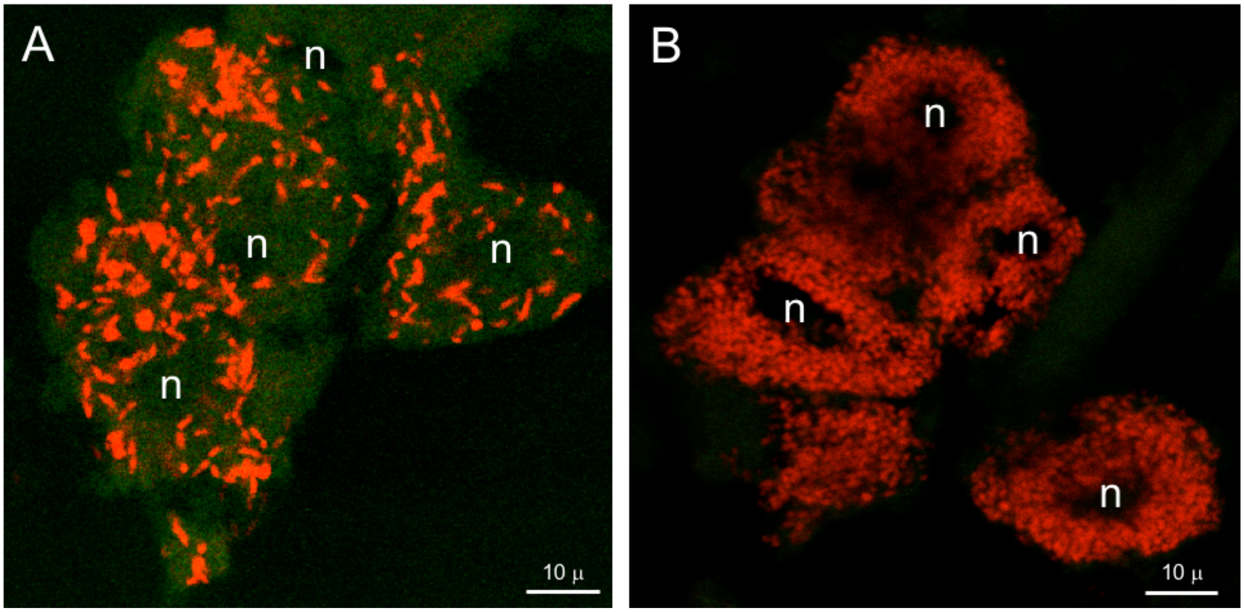

Supplementary Figure 6. Localization of bacteriocyte-associated endosymbionts by FISH in *Adelges lariciatus* and *Adelges abietis*. **A.** FISH performed on third-instar stages of *Adelges lariciatus* to detect '*Ca*. Vallotia" using specific probe b125 labeled with Alexa-568-5-dUTP (red). **B.** FISH performed on third-instar stages of *Adelges abietis* using general eubacterial probe 1507r labeled with Alexa-568-5-dUTP (red); both '*Ca*. Vallotia" and '*Ca*. Profftia' symbionts are localized with this general probe. Green, autofluorescence of insect cuticle; n, bacteriocyte nucleus.

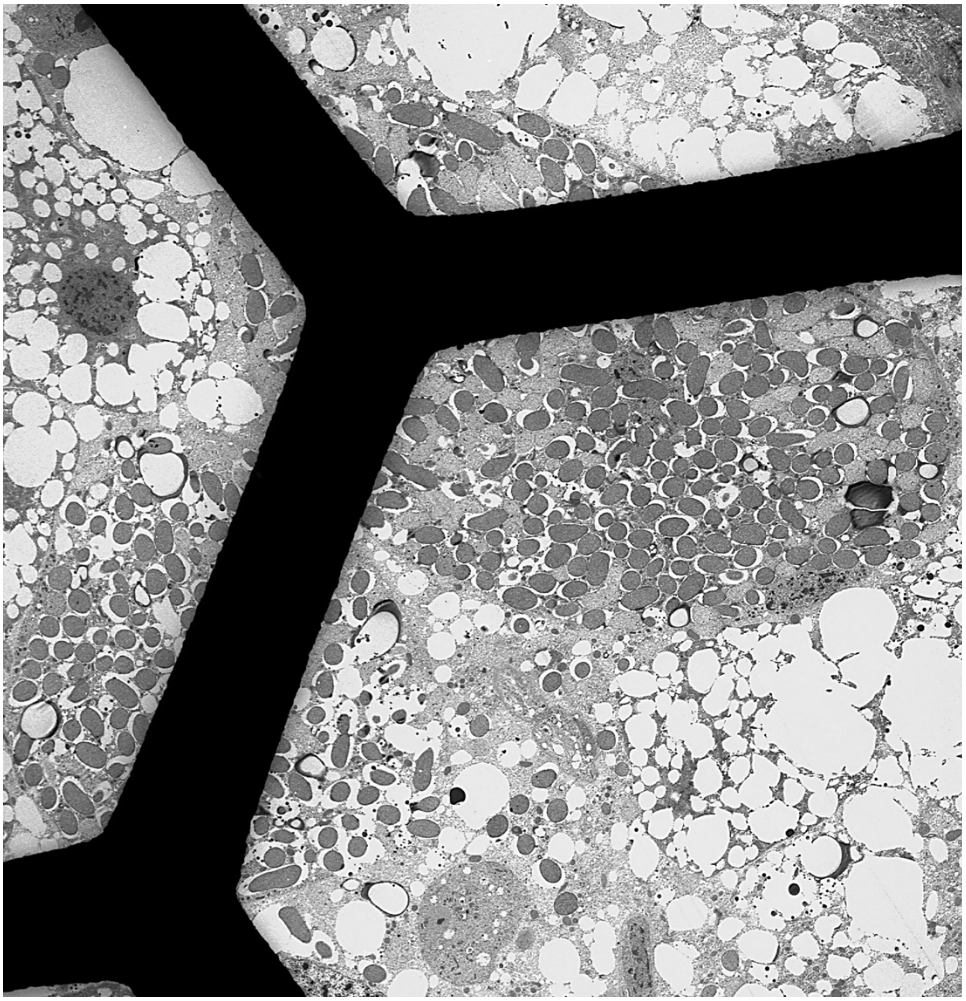

Supplementary Figure 7. Ultrastructure of *Adelges abietis* endosymbionts residing in bacteriocytes. Ultrathin section of bacteriome in whole-mount insects, showing two distinct bacteriocytes containing two bacterial morphotypes, one light-staining and one dark-staining, corresponding to '*Ca*. Vallotia" and '*Ca*. Profftia' symbionts.

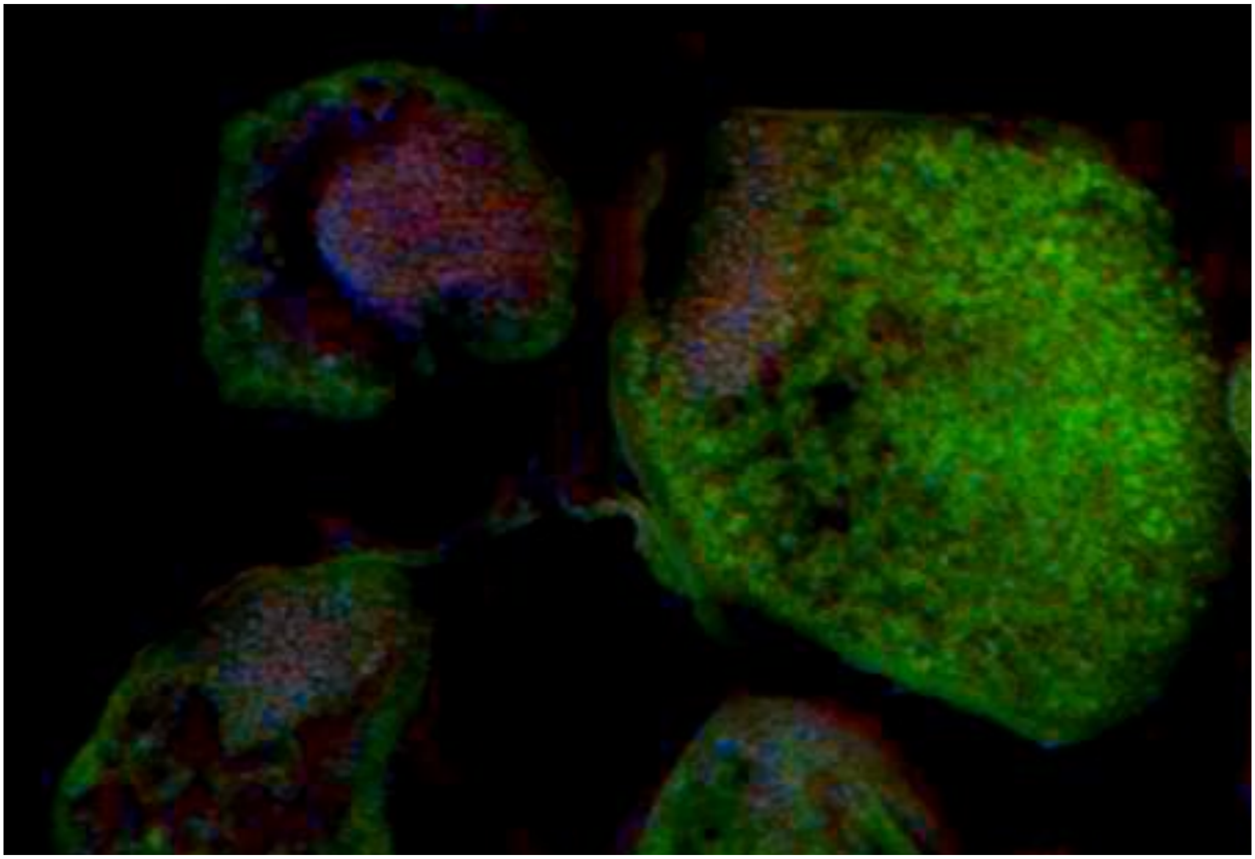

Supplementary Figure 8. Localization of bacteriocyte-associated endosymbionts in eggs from adult *P. similis* by FISH. Simultaneous detection of '*Ca*. Annandia pinicola' using GamC-440 labeled with Bodipy-650-14-dUTP (blue), and '*Ca*. Hartigia pinicola' using PinGam2-470 and PinGam2-828 labeled with Alexa-568-5-dUTP (red). Green, autofluorescence of insect cuticle.
